# Retrospective and projected warming-equivalent emissions from global livestock and cattle calculated with an alternative climate metric denoted GWP*

**DOI:** 10.1371/journal.pone.0288341

**Published:** 2023-10-02

**Authors:** Agustin del Prado, Brian Lindsay, Juan Tricarico

**Affiliations:** 1 Basque Centre for Climate Change (BC3), Edificio Sede no. 1, Planta 1, Parque Científico de UPV/EHU, Barrio Leioa, Bizkaia, Spain; 2 Ikerbasque—Basque Foundation of Science, Bilbao, Spain; 3 Global Dairy Platform, Rosemont, IL, United States of America; 4 Innovation Center for U.S. Dairy, Rosemont, IL, United States of America; Sichuan University, CHINA

## Abstract

Limiting warming by the end of the century to 1.5°C compared to pre-Industrial times requires reaching and sustaining net zero global carbon dioxide (CO_2_) emissions and declining radiative forcing from non-CO_2_ greenhouse gas (GHG) sources such as methane (CH_4_). This implies eliminating CO_2_ emissions or balancing them with removals while mitigating CH_4_ emissions to reduce their radiative forcing over time. The global cattle sector (including Buffalo) mainly emits CH_4_ and N_2_O and will benefit from understanding the extent and speed of CH_4_ reductions necessary to align its mitigation ambitions with global temperature goals. This study explores the utility of an alternative usage of global warming potentials (GWP*) in combination with the Transient Climate Response to cumulative carbon Emissions (TCRE) to compare retrospective and projected climate impacts of global livestock emission pathways with other sectors (e.g. fossil fuel and land use change). To illustrate this, we estimated the amount and fraction of total warming attributable to direct CH_4_ livestock emissions from 1750 to 2019 using existing emissions datasets and projected their contributions to future warming under three historical and three future emission scenarios. These historical and projected estimates were transformed into cumulative CO_2_ equivalent (GWP_100_) and warming equivalent (GWP*) emissions that were multiplied by a TCRE coefficient to express induced warming as globally averaged surface temperature change. In general, temperature change estimates from this study are comparable to those obtained from other climate models. Sustained annual reductions in CH_4_ emissions of 0.32% by the global cattle sector would stabilize their future effect on global temperature while greater reductions would reverse historical past contributions to global warming by the sector in a similar fashion to increasing C sinks. The extent and speed with which CH_4_ mitigation interventions are introduced by the sector will determine the peak temperature achieved in the path to net-zero GHG.

## Introduction

The Intergovernmental Panel on Climate Change [1] estimated that the average temperature of the Earth’s atmosphere and oceans increased by approximately 1.1°C from 1850 to 2020. As part of the global effort to avoid dangerous consequences of climate change, the Paris Climate Agreement sets out a global framework to limit global warming to well below 2°C and pursue efforts to limit it to 1.5°C compared with preindustrial levels by 2050 [2]. In order to avoid surpassing these temperature limits, the article 4 of the Paris Agreement sets a greenhouse gas (GHG) mitigation goal of achieving ‘a balance between anthropogenic emissions by sources and removals by sinks of GHGs‘. One of the interpretations is that this balance can be reached with net-zero CO_2_-equivalent emissions [3].

‘Net-zero‘, is defined in the Working Group I contribution to the Sixth Assessment Report of the IPCC [1] for both carbon dioxide (CO_2_) emissions (denoted as net-zero CO_2_ or C neutrality) and for all GHG species (denoted as net-zero GHG or GHG neutrality). Net-zero CO_2_ (in brackets for GHG) is the condition in which (metric-weighted) anthropogenic CO_2_ (GHG) emissions associated with a reporting entity are balanced by (metric-weighted) anthropogenic CO_2_ (GHG) removals over a specified period [1]. Temperature outcomes relative to net-zero GHG emissions will vary with the metric chosen to compare emissions and removals of different GHG [1].

Emission metrics provide a means of comparing different GHG emissions and removals by placing them on the same scale. This is typically done by quantifying a specified climate impact of a non-CO_2_ gas relative to that of a CO_2_ emission and is reported as ‘CO_2_-equivalents’ [4]. The most common GHG emission metric is the 100-year global warming potential (GWP_100_) that compares the radiative forcing accumulated over a user-defined time-horizon (100 years) resulting from a pulse-emission of a specific GHG to a pulse-emission of an equal mass of CO_2_ [4]. The development of alternatives referred to as ‘step-pulse’ emission metrics, such as CGTP [5] or GWP* [6], arises from the need to account for differences between the effects of long and short-lived GHGs on global temperature change [7]. Whereas methane (CH_4_)’s impacts on temperature varies strongly with time after emissions occur due to its short atmospheric life, CO_2_’s impact on temperature remains relatively constant for hundreds of years after the emission occurs [7]. Also, CO_2_, once emitted, leads to increasing global temperature until net-zero CO_2_ emissions are reached. By contrast, reductions in CH_4_ emissions lead to reversing warming within a few decades. These differences in temperature change are hidden when calculating and using annual CO_2_-e emissions to describe the impact of mitigation and targets based on aggregated annual emission rates [7]. In fact, GWP* was not developed to capture this behaviour, which was already well known, but to demonstrate that it could be quantified relatively simply while continuing to use the already familiar GWP concept [7].

It is recognized that GWP_100_ shows the relative climate effect at one point in time resulting from an emission without needing comparison with past emissions [4] and is therefore inaccurate when estimating warming associated with emission time-series or pathways. This was highlighted by IPCC [1], which noted how GWP_100_ either overestimates or underestimates global surface temperature changes depending on whether the CH_4_ emissions rate was constant (overstated by a factor of 3–4) or increasing at high rates (understated by a factor of 4–5) over a 20-year time horizon [8]. Step-pulse metrics like GWP* can directly illustrate the anticipated temperature changes resulting from different emission pathways and incorporate them in ‘cumulative emission budgets’ or ‘C budgets’ to estimate how much each non-CO_2_ gas contributes to warming [9] or remaining C budgets [10].

The agriculture sector is a large contributor of CH_4_ emissions through rice cultivation and enteric fermentation and manure management from livestock. Livestock and global cattle (as defined by dairy and beef cattle and buffalo) contribute 30% and 24% of the global anthropogenic CH_4_ emissions, respectively [11]. Therefore, the choice of metric will strongly affect the calculated impact from CH_4_ mitigation by the livestock and global cattle sectors as well as their contributions to historical warming from past emissions compared to CO_2_ emission sources. These considerations could have substantial implications for climate goals, practice and policies recommendations, and their evaluation.

This article explores the impacts of acknowledging the differences between CH_4_ and CO_2_ using warming-equivalent emissions as calculated by GWP*. In concrete terms, we aimed to: (i) illustrate the use of GWP* instead of GWP_100_ to compare the impact of CH_4_ and CO_2_ emission pathways on atmospheric warming using emissions from global fossil fuel CO_2_, land use change CO_2_ and livestock CH_4_ reported in the scientific literature, (ii) investigate, using these metrics, the retrospective warming added by CH_4_ emissions from livestock in aggregate and from global cattle (as defined by dairy and beef cattle and buffalo) globally between 1750–2019 and 1961–2019, respectively, and (iii) analyse, using these metrics, the implications on future global temperature change of adopting different CH_4_ mitigation strategies that lead to warming stabilisation by the global cattle industry.

## Materials and methods

### Sources of emissions used for the retrospective analysis

The study uses several existing sources of CH_4_ emissions data to illustrate the use of the GWP* climate metric to calculate warming-equivalent emissions and global temperature change from historical livestock and global cattle numbers. It must be noted that we used total livestock (including all animal species) CH_4_ emissions for the long-term retrospective analysis (1750–2019) since data disaggregating livestock emissions by species (i.e. cattle) were not available for the years before 1961. For the short-term analysis (1961–2019) we used historical cattle (dairy and beef) and buffalo CH_4_ emissions (all together referred as ‘global cattle’).

Long-term (1750–2019) historical livestock annual enteric and manure CH_4_ emissions data were obtained from:

- Reisinger and Clark [12] (1750–2009)
- EDGAR database https://edgar.jrc.ec.europa.eu/ (accessed on March 20, 2022) (2010–2019)

Short-term (1961–2019) historical cattle and buffalo annual enteric and manure CH_4_ emissions were obtained from the FAOSTAT database [11]. For simplicity purposes, ‘global cattle’ refers to cattle (dairy and beef) and buffalo in this study.

A dataset comprising long-term (1750–2019) historical annual CO_2_ emissions from fossil fuel (excluding carbonation) and land use change (LUC) from [13] was used for long-term (since 1750) historical comparisons with livestock CH_4_ emissions and short-term (e.g. since 1961) historical comparisons with global cattle CH_4_ emissions.

As mentioned above, long-term historical emissions data (including livestock) were considered since 1750 mainly because Friedlingstein et al. [13] includes data from this year which coincides with the beginning of the industrial revolution. In contrast, Reisinger and Clark [12] assumed no livestock emissions prior to 1860 in their study for practical reasons. Different alternative assumptions for livestock CH_4_ emissions rates were simulated (details are explained in the scenario testing section below) to estimate the potential effects on global temperatures of including or excluding livestock emissions during the 1750–1859 period.

### Description of the data used for the retrospective analysis

**Fig 1:**
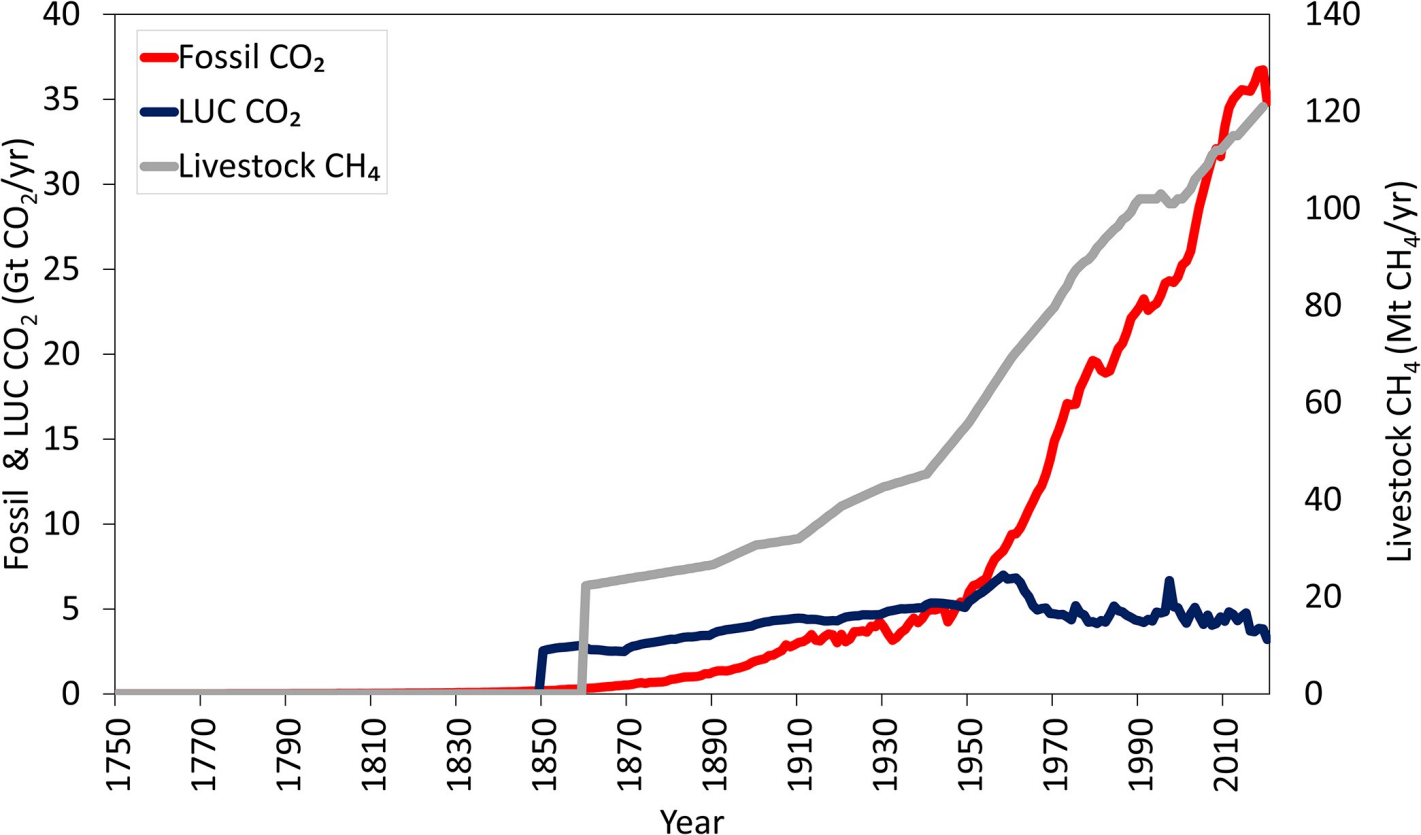
Historical annual global emissions from fossil CO_2_ use excluding carbonation (Fossil CO_2_) and from land use change (LUC CO_2_) (Gt CO_2_ per year) and global CH_4_ emissions from livestock (Mt CH_4_ per year). Note that CO_2_ and CH_4_ results are expressed in different units and each refers to the left and right y axes, respectively. Data source: CO_2_: Friedlingstein et al. (2021) and CH_4_: Reisinger and Clark (2018) (1750–2009) and EDGAR database (2010–2019).

The historical dataset used in our study shows that fossil CO_2_ emissions increased steadily, especially since the mid-twentieth century, to approximately 36.7 Gt CO_2_/yr by 2019 (Fig 1). In contrast, CO_2_ emissions from LUC peaked in 1958 (7 Gt CO_2_/yr) and 1997 (6.7 Gt CO_2_/yr) but dropped during the last two decades to approximately 3.2 Gt CO_2_/yr by 2019 (Fig 1). The dataset used in our study shows that long-term historical livestock CH_4_ emissions increased from 22 to 121 Mt CH_4_/yr from 1860 to 2019 (Fig 1).

**Fig 2:**
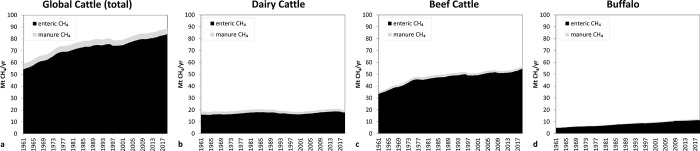
Methane emissions from global cattle (aggregating dairy and beef cattle and buffalo) (a), dairy cattle (b), beef cattle (c) and buffalo (d) for both enteric and manure sources. Results are expressed as Mt CH_4_ per year. Data source: FAOSTAT.

The dataset used for short-term (1961–2019) historical global cattle CH_4_ emissions shows they increased from approximately 59 Mt CH_4_/yr in 1961 to almost 89 Mt CH_4_/yr by 2019 (Fig 2A). In this dataset, if we disaggregate the data by animal types and sources, beef cattle represent the largest share of CH_4_ emissions from the global cattle sector over 1961 to 2019 (Fig 2C), followed by dairy cattle (Fig 2B) and buffalo (Fig 2D). Enteric emissions represent approximately 94% of total CH_4_ output by the global cattle sector with the remainder coming from manure management. The enteric contribution to total global cattle CH_4_ emissions has grown from approximately 92% in 1961 to 95% in the last decade. The largest increases in CH_4_ emissions occurred in the 1960s and 70s followed by stable emissions in the 90s and a more modest increase in the 2000s.

### CO_2_-e and CO_2_-we associated to using GWP_100_ or GWP* to estimate global temperature changes

Cumulative CO_2_ emissions show a near-linear relationship with their induced global warming [14]. This proportionality is represented by a coefficient that is referred to as TCRE (Transient Climate Response to cumulative carbon Emissions). This TCRE value can be multiplied by cumulative CO_2_ emissions to obtain an estimate of temperature change due to the CO_2_ burden experienced [15]. According to IPCC (2021) [1], each Tt of cumulative CO_2_ emissions is assessed to likely cause a 0.27°C to 0.63°C increase in global surface temperature with a best estimate of 0.45°C.

For other non-CO_2_ GHGs, a relationship between the cumulative amount emitted and their induced global warming can only be approximated if the GHG is a long-lived gas such as CO_2_ (e.g. nitrous oxide: N_2_O) and when the gas is expressed as CO_2_-e using the GWP_100_ metric [13]. For GHGs with short-life in the atmosphere (i.e. CH_4_), a new climate metric was developed, denoted GWP* with the associated CO_2_ warming-equivalent (CO_2_-we), to provide a direct link between calculated cumulative CO_2_-we emissions and global warming via the TCRE value [16].

The GWP* metric was applied in this study to calculate CO_2_-we emissions for CH_4_ sources from livestock and global cattle. The following equation, developed by Smith et al. [17] that is adaptable to calculate CO_2_-we for all short-lived GHG on a particular year, was used:

CO2we=4.53×E100(t)−4.25×E100(t−20) (1)

Where, E100 corresponds to conventional global warming CO_2_-equivalent emissions calculated using GWP_100_. This value is required for both the emission rates for the current year, *E*100 (*t*), and from 20 years ago, *E*100 (*t*−20). When there is a large difference between these two emission rates, a large CO_2_-we value is returned, emphasizing the significant and rapid impact of changing methane emission rates.

Emissions are used from years t (the year for which CO_2_-we emissions are being calculated) and from t-20 (20 years prior). This allows GWP* to represent the impact of new emissions (which cause strong additional warming), stable emissions (minimal additional warming) and reducing emissions (which reverses some past warming) [6].

Methane emissions were converted into CO_2_-e using the value indicated by the IPCC report AR6 of GWP_100_ = 27.2 ([1]).

The following steps and calculations were performed to estimate the induced global warming (global temperature change) by the different global emissions sources from a particular period:

- Selection of emissions time series which is defined by an initial year (e.g. 1750, 1961, or 2020) and an ending year (e.g. 2019, 2050, or 2100).
- Calculate the cumulative emissions across the study period. For CH_4_, it is required that emissions are expressed as CO_2_-we emissions following Eq 1. Although not applicable in our study, other non-CO_2_ GHG sources like N_2_O would require that emissions are expressed as CO_2_-e emissions using GWP_100_. For CO_2_ emission sources, no conversion is required.
- Multiply the value of cumulative emissions by the TCRE value to obtain global warming induced in the study period by these emissions. For this study, although the TRCE value of 0.45 K°/Tt CO_2_ [1] is used throughout all scenarios, an alternative value of 0.4 K°/Tt CO_2_ (used by Lynch et al. [8]) is used for the long-term historical livestock CH_4_ emissions series as an illustration of the sensitivity of the final global warming result to changes in the choice of the TRCE value.

### Scenarios for testing the framework to estimate the impact of GHG emissions on global temperature change

Different scenarios were developed and shown in Table 1 to illustrate the usage of GWP* for retrospective time-series analysis (1–3) and for forecasting future contributions to warming from different emission sources (4–5):

1. Comparison between global warming induced by long-term (1750–2019) historical emissions of fossil CO_2_, LUC CO_2_, and livestock CH_4_ according to the following assumptions:
  - no livestock emissions during 1750–1859 (as assumed by Reisinger and Clark (2018) [12])
  - annual livestock emissions in 1750 were half of 1860 considering that the global human population was approximately half and had, and increased gradually up to 1860 (this scenario also assumes a non-changing meat and milk consumption per capita)
  - annual livestock emissions were similar to 1860 during the 1750–1860 period.

**Table 1:** Overview of the set-up of the different scenarios for testing the framework to estimate the impact of GHG emissions on global temperature change.

Scenario	Analysis type	length of period	warming basis	reference year (s)	target year	Sectors involved
**1**	retrospective (past)	long-term	marginal	1750	2019	livestock CH_4_, fossil CO_2_, LUC CO_2_
**2**	retrospective (past)	short-term	additional	1981, 1990, 2000, 2010	2019	Global cattle CH_4_, fossil CO_2_, LUC CO_2_
**3**	retrospective (past)	short-term	additional	1981, 1990, 2000, 2010	2019	Global cattle CH_4_
**4**	forecasting (future)	long-term	marginal	1750	2100	livestock CH_4_, fossil CO_2_, LUC CO_2_
**5**	forecasting (future)	short-term	additional	1990	2050	Global cattle CH_4_

‘Marginal warming’ and ‘Additional warming’ denote the warming that emissions causes, relative to the absence of that emission and a pre-existing level of emissions at the reference year, respectively [1,18].

It must be noted that, the ‘global warming induced’ calculated in this scenario reports the global temperature change since pre-industrial years or, in other words, the absolute level of global warming attributable to these sectors and assumed as anthropogenic nowadays. By including a period that goes back to preindustrial time, we could explore warming impacts relative to a ‘reference condition’ of no anthropogenic emissions (marginal warming). Setting the reference time in this scenario to preindustrial times reflects the type of information provided by the GWP_100_ (marginal warming-basis), but retains a dynamic component in the case of GWP* [18]. The difference between marginal and ’additional’ warming, as measured by GWP* in the manner it is mostly applied, is explained in detail in the IPCC WGIII contribution to the AR6 Report, particularly in supplementary material to chapter 2 ([1]).

2. Comparison between global warming induced by short-term (1961–2019) historical emissions of fossil CO_2_, LUC CO_2_, and global cattle CH_4_ for the following periods: 1981–2019, 1990–2019, 2000–2019, and 2010–2019.

It must be noted that, the ‘global warming induced’ calculated as in this scenario, reports the temperature change over time, relative to a reference level of warming caused by prior emissions up to the beginning of the time series being evaluated.

3. Comparison between cumulative CO_2_ equivalent emissions expressed as CO_2_-we (using GWP*) vs. CO_2_-e (using GWP_100_) for short-term (1961–2019) historical global cattle CH_4_ emissions for the following periods: 1981–2019, 1990–2019, 2000–2019, 2010–2019.

This comparison will be useful to estimate the contribution of CH_4_ emission rates affecting the remaining carbon budget (as expressed by cumulative CO_2_-we emissions) and hence, the error that would imply expressing CH_4_ as cumulative CO_2_-e emissions in C budget calculations. The C budget is defined as the cumulative amount of CO_2_ emissions, up to net-zero, that would be consistent with limiting warming to a specified level while considering the contribution of non-CO_2_ climate forcers to total warming [19].

4. Comparison of projected global warming associated with future emission pathways for global fossil CO_2_, LUC CO_2_, and livestock CH_4_ emissions (2020–2100 period) for 3 different emission rates assumptions:
  - keeping emissions unchanged from 2020
  - reducing global GHG emissions in a sustained way (1% decrease per year, constant over time)
  - reducing global GHG emissions up to reaching 0 emissions (net-0 CO2 or net-0 CO_2_-e using GWP_100_ for livestock CH_4_) by 2100 by reducing fossil CO_2_: 0.44 Gt CO_2_/yr; LUC CO_2_: 0.04 Gt CO_2_/yr and livestock CH_4_: 1.5 Mt CH_4_/yr.

In these projected scenarios, warming associated to future emission pathways will include the estimated warming legacy from 1750. Hence, warming results will refer to absolute temperature levels attributable to each sector since the industrial revolution started.

5. Analyzing the projected global warming associated with 3 futures global CH_4_ cattle emission pathways leading to a stabilisation of global temperatures at year 2050 (1990–2050 period) varying in the time and intensity when absolute CH_4_ emission rates are reduced as follows:
  - fast: large reductions (greater than those required for sustained emission rate reductions: 0.5% decrease per year) in global cattle CH_4_ emissions are introduced during the first decades (2020–2040) followed by reductions at smaller rates (2040–2050) (0.1% decrease per year).
  - sustained: reductions in global cattle CH_4_ emissions in a sustained way starting in 2020 (0.32% decrease per year, constant over time)
  - delayed: increase in absolute emission rates for the first 2 decades (2020–2040) (0.25% increase per year), followed by large reductions (0.84% decrease per year)

in global cattle CH_4_ emissions are introduced during the last decade (2040–2050).

Temperature stabilisation is achieved in the year 2050 when CH_4_ emissions expressed as CO_2_-we using GWP* reach the value of zero (0). It must be noted that reaching zero (0) CO_2_-we emissions at a particular year can be assumed analogous to the concept of stabilizing temperatures by reducing CO_2_ emissions when reaching net-zero CO_2_ emissions [20]. Aggressive CH_4_ mitigation could reverse much of the CH_4_-induced warming experienced at a particular year, having an analogous temperature impact to actively removing past CO_2_ emissions (e.g. by reforesting), and hence reported as a negative CO_2_-we (i.e. CO_2_-we/yr <0). It must be noted that the ‘global warming’ calculated in these scenarios reports the temperature change over time relative to a reference level of warming caused by prior emissions up to the beginning of the time series being evaluated.

## Results and discussion

### Global warming induced by long-term (1750–2019) historical emissions of fossil fuel CO_2_, LUC CO_2_, and livestock CH_4_

**Fig 3:**
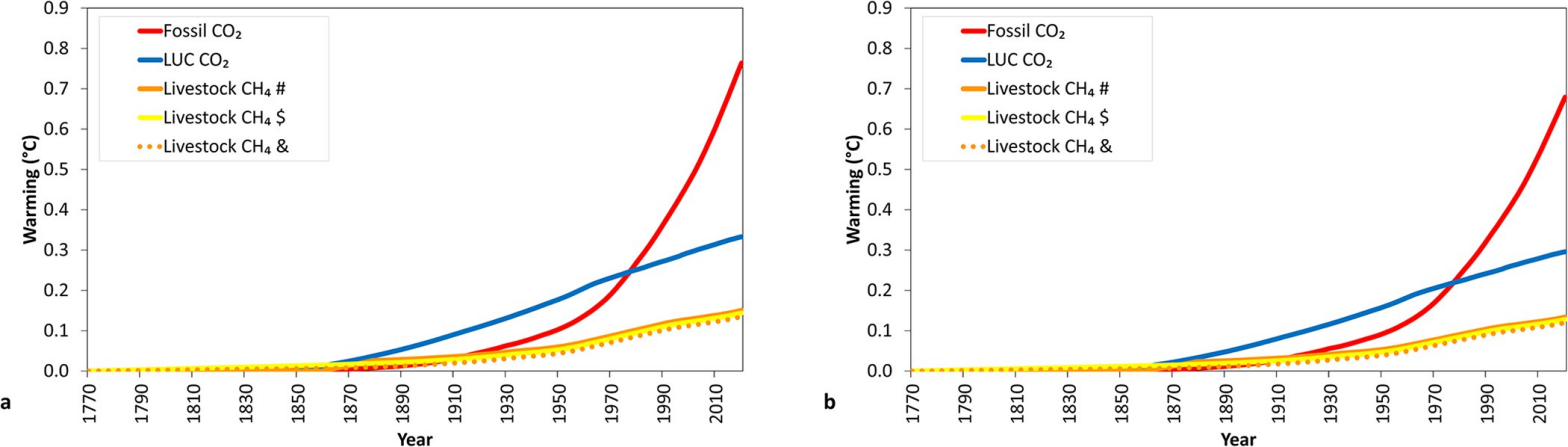
Historical warming impact of global fossil CO_2_ emissions excluding carbonation (Fossil CO_2_), global land use change (LUC CO_2_) emissions and livestock CH_4_ as estimated by multiplying aggregated CO_2_ emissions and CO_2_-we (using GWP*) (for CH_4_ sources) emissions by the TCRE value: (a) 0.45°K/Tg CO_2_ and (b) 0.40°K/Tg CO_2_. For livestock, there are 3 different assumptions of CH_4_ emission rates from global livestock in the period between 1750–1860: # and solid orange line: 0 CH_4_/yr emissions, $ and yellow line: Sustained increased emissions from 11.5 to 22.3 kt CH_4_/yr and & and dotted orange line: 22.3 kt CH_4_/yr during 1750–1860.

Our results show that the estimated global warming associated with fossil fuel CO_2_, LUC CO_2_, and livestock CH_4_ cumulative emissions from 1750 to 2019 were 0.75°C, 0.33°C and 0.15°C, respectively (Fig 3A). This represents total global warming of 1.23°C corresponding to cumulative emissions of 1663 Gt CO_2_ from fossil fuel, 737 Gt CO_2_ from LUC, and 9.5 Gt CH_4_ from livestock that equals 258 Gt CO_2_-e when using GWP_100_ and 333 Gt CO_2_-we when using GWP* in the calculations. As expected, using the lower TRCE value of 0.4 K°/Tt CO_2_ (compared with 0.45) results in a proportional reduction in estimated global warming values to 0.67°C, 0.29°C and 0.13°C for fossil fuel CO_2_, LUC CO_2_, and livestock CH_4_ emissions, respectively (Fig 3B). The total global warming estimated in this study for the 1750–2019 period (1.23°C) is comparable to total human-caused global surface temperature increases from 1850–1900 to 2010–2019 of 0.8°C-1.3°C reported by IPCC (2021) [1]. Our estimates for global livestock-induced warming (0.15°C) are slightly greater than those reported (0.11°C) using the MAGICC climate model for the slightly shorter period 1750–2010 [12].

Considering that livestock existed for many centuries before the industrial revolution began, the scenarios (1.ii and 1.iii) that included livestock emissions between 1750 and 1859 led to lower historical contributions to warming (0.12–0.13°C, Fig 3A) than the zero livestock emissions scenario (1.i). When livestock emissions were set to zero prior to 1860, the GWP*-TCRE calculation estimates warming on a marginal-basis, in other words the temperature changes are compared to those emissions not occurring. In this sense, using the GWP*-TCRE calculation on those scenarios that reflect livestock CH_4_ emissions prior to 1750, will allow us to consider the level of CH_4_ emissions already existing in 1750 (prior to the reference time we generally account for the antropogenic emissions in the IPCC-based frameworks) and consequently, allow us to adjust the warming that livestock CH_4_ has added since the industrial revoluition (reference time). At the country level, although a comparison of the current anthropogenic livestock CH_4_-induced warming with the pre-industrial reference will generally show a large net warming due to much higher current CH_4_ emission for some specific countries (e.g. Germany). CH_4_ estimates suggest that, compared with XIX century, Germany’s livestock has been emitting less enteric CH_4_ since 2003 [21].

Table 2 shows the estimated additional warming attributed to global CO_2_ (fossil and land use change) and livestock CH_4_ for three different periods: 1770–1970, 1970–1990, and 1990–2019. The additional warming contributed by all 3 sources during the first 200 years (0.51°C for 1770–1970) is similar to the additional warming contributed during the last 3 decades (0.49°C for 1970–2019). However, CO_2_ from fossil sources contributes the vast majority, at more than 80% of the of the total warming over the past three decades. In fact, the relative contribution of fossil fuel emissions to global warming accelerated over time while those from LUC and global livestock decreased over time with Sanderman et al. [22] reporting similar findings. In contrast, the contribution by LUC CO_2_ was particularly severe prior to 1970 accounting for 45% of the additional warming associated with these three sources of global emissions. The contribution to global warming by livestock CH_4_ represented 17% from the three sources examined up to 1970 and shrunk to 7% in the last three decades (1990–2019). The historical contributions to warming from each source are quite different. LUC contributions peaked by 1970 and are now decreasing. Fossil fuel contributions always increased since the start of the industrial revolution and accelerated dramatically since the mid-20th century. Livestock contributions increased throughout the period studied but at a much lower rate than fossil fuel contributions.

**Table 2:** Additional temperature from fossil CO_2_ emissions excluding carbonation, land use change (LUC) CO_2_ and livestock CH_4_ for different periods (1770–1970; 1970–1990; 1990–2019) and % of this temperature within each period that can be attributed to each sector.

	Additional temperature (°C)	Fossil CO_2_	LUC CO_2_	Livestock CH_4_
**1770–1970**	0.51	37%	45%	17%
**1970–1990**	0.24	71%	17%	12%
**1990–2019**	0.49	81%	12%	7%

### Global warming induced by short-term (1961–2019) historical emissions of fossil fuel CO_2_, LUC CO_2_, and global cattle CH_4_

**Fig 4:**
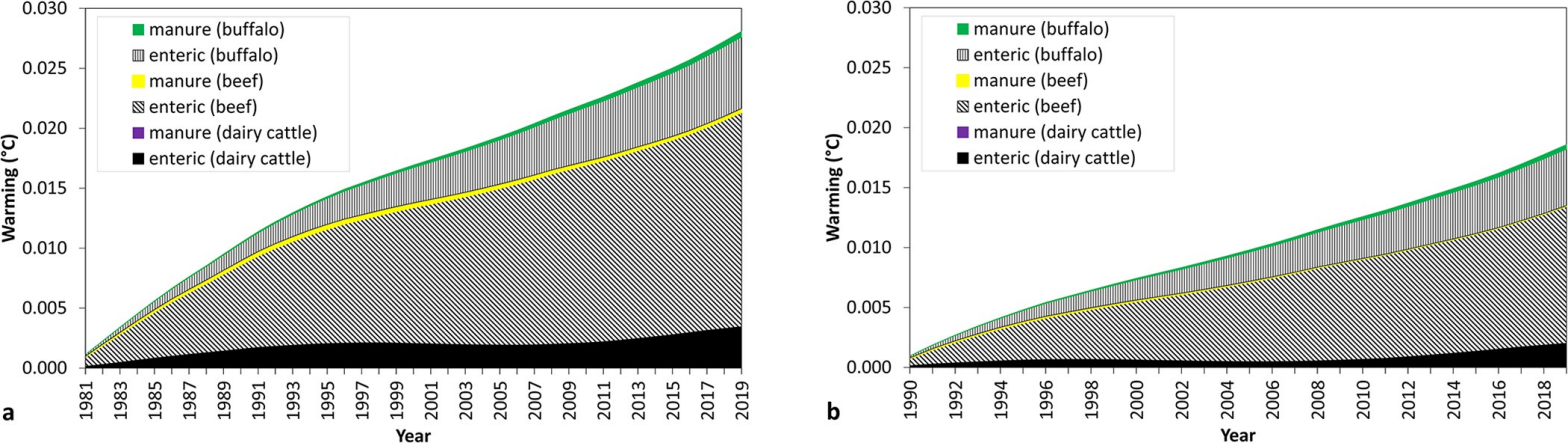
Additional warming associated with methane emissions from different sources (enteric and manure) of global cattle systems (dairy, beef and buffalo) until 2019 and since 1981(a) and 1990 (b). Additional warming is calculated by multiplying cumulative CO_2_-we (using GWP*) emissions with the TCRE value (0.45°K/Tg CO_2_-we).

Short-term global warming estimates associated with global cattle CH_4_ emissions (dairy cattle, beef cattle and buffalo) were 0.028°C for 1981–2019 and 0.019°C for 1990–2019 (Fig 4). For both periods, when animal types are dissagregated, we found that the largest additional global warming can be attributed to enteric CH_4_ from the beef cattle sector followed by enteric CH_4_ from the buffalo and dairy cattle sectors (Fig 4).

**Fig 5:**
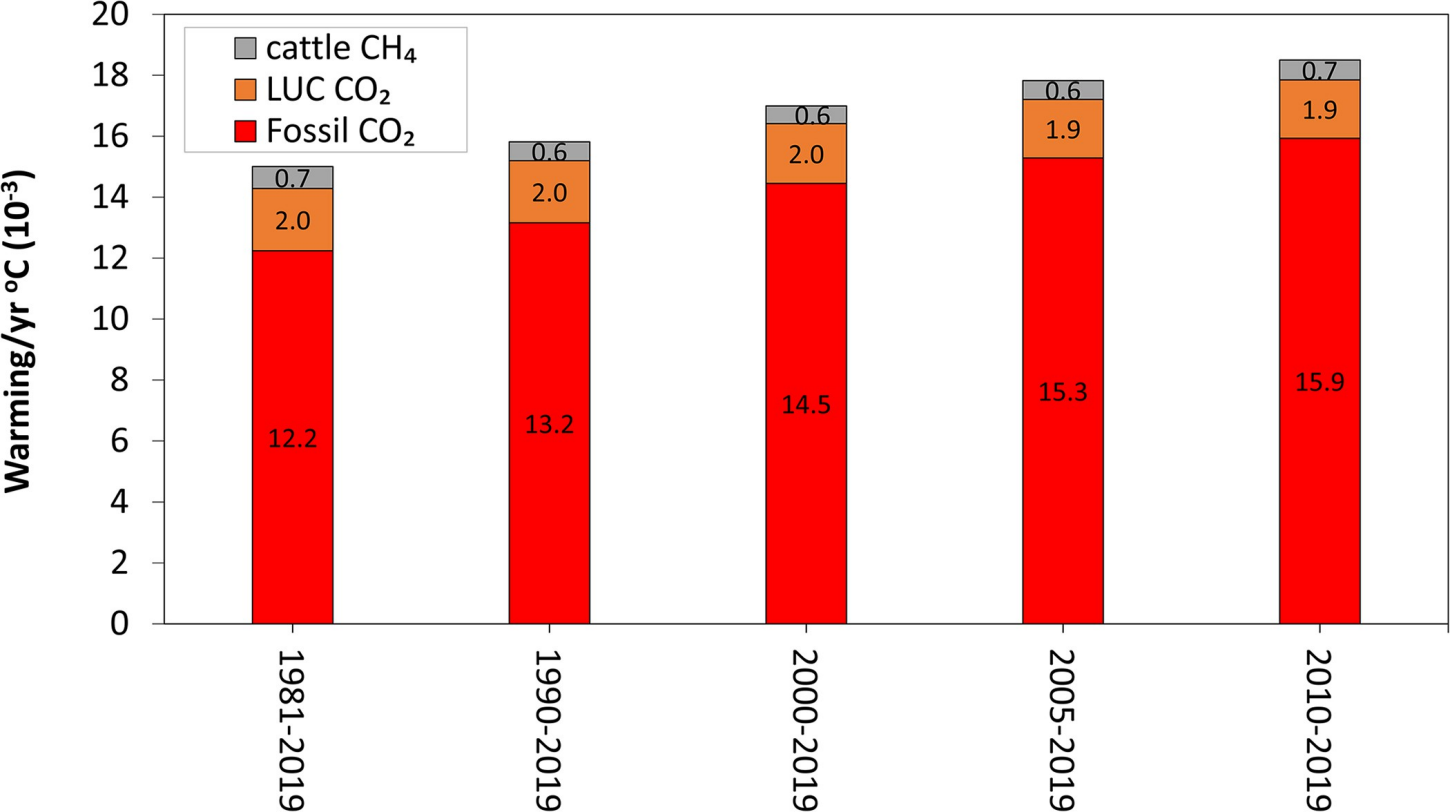
Averaged annual additional warming (as calculated by dividing the additional warming within the analysed period by the number of years in such period) associated with global fossil fuel CO_2_, land use change CO_2_ and global cattle CH_4_ for periods with different reference years (1981, 1990, 2000, 2005 and 2010) until 2019.

Fig 5 shows that the contribution of emissions from fossil fuel to historical warming from 1981 to 2019 is 12-fold greater than from global cattle CH_4_ and 6-fold greater than from LUC. Also, the additional warming per year associated to global cattle CH_4_ during this period is in the range of 4 to 6% compared with the additional warming attributed to fossil fuel CO_2_ emissions. This is a significant difference that provides perspective on the contributions from the three different GHG emission sources. Moreover, the averaged annual additional warming associated to fossil CO_2_ emissions, which expresses the relative annual added temperature (as calculated by dividing the additional warming within the analysed period by the number of years in such period), became greater in more recent years (from 12 m°C/yr in 1981–2019 to 16 m°C/yr in 2010–2019).

Although not explored in this study, when the approach is applied to emission assessments at sub-global scale (e.g. country-level: [23–25]), its applicability, similarly as with CO_2_ ([16]), should carefully consider potential equity impacts ([26]). For example, Reisinger et al. [19] suggest having a clear separation of legacy warming from past emissions, which is significant only for long-lived gases, and marginal warming from current and future emissions and removals, which applies for all gases, to best address mitigation strategies. Nevertheless, how sub-global scale entities, such as countries, might incorporate the warming from past CO_2_ or CH_4_ emissions into climate policy poses questions that go beyond the scope of this study.

### Comparison between cumulative CO_2_ equivalent emissions expressed as CO_2_-we (using GWP*) *vs*. CO_2_-e (using GWP_100_) for short-term (1961–2019) historical global cattle CH_4_ emissions for the following periods: 1981–2019, 1990–2019, 2000–2019, 2010–2019

**Fig 6:**
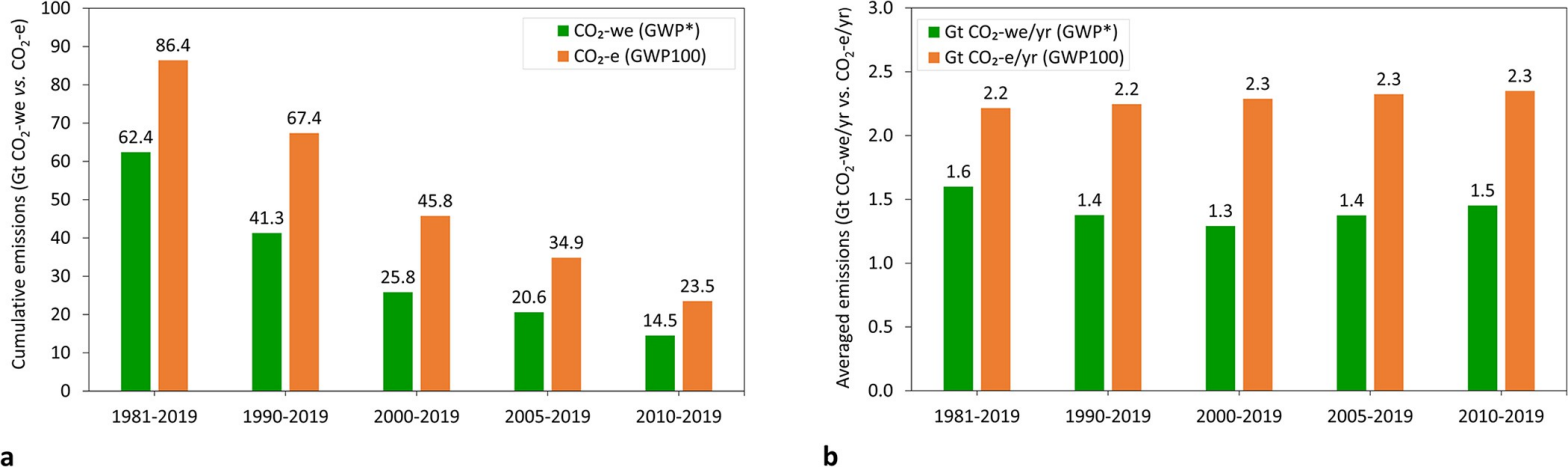
Cumulative emissions (a) and average annual emissions (as calculated by dividing the cumulative emissions within the analysed period by the number of years in such period) (b) as expressed using GWP* (CO_2_-we) and conventional GWP_100_ (CO_2_-e) coming from methane emissions from global cattle systems for different reference years (1981, 1990, 2000, 2005 and 2010) until 2019. (white: CO_2_-we and grey: CO_2_-e). Note that axes from Fig 6A and 6B have different magnitudes.

Cumulative (Fig 6A) and average annual emissions (CO_2_ equivalent emissions per year) expressed as CO_2_-we (using GWP*) and CO_2_-e emissions (using GWP_100_) associated to global cattle CH_4_ emissions varied depending on length of period assessed. As expected, cumulative emissions expressed as CO_2_-e increase linearly with longer assessment periods, but when expressed as CO_2_-we, the changes in cumulative emissions responded to the rate of change of CH_4_ emissions. Therefore, cumulative CO_2_-we values were lower than if reported as CO_2_-e by 38% on average across all periods examined (Fig 6A), but the differences ranged from 28% (1981–2019) to 44% (2000–2019) depending on how CH_4_ emission rates changed in the years included in each period. This observation implies that each tonne of global cattle CH_4_ emissions contributed less additional warming than that from 27.2 tonnes of any source of CO_2_ (27.2 is methane’s GWP_100_ value) in all the periods examined. In fact, increasing the rate of CH_4_ emissions has a greater impact on global mean surface temperature per tonne of CH_4_ emitted than constant CH_4_ emissions, while CO_2_ emissions have the same impact on global mean surface temperature per tonne of CO_2_ emitted regardless of emission trajectory [6].

Three other studies that compared CH_4_ emission pathways expressed as CO_2_-e using GWP_100_ and CO_2_-we using GWP* found similar results. Del Prado et al. [27] estimated additional warming associated to historical direct GHG emissions from dairy small ruminants in continental Europe. These authors report that the European sheep and goat dairy sector did not contribute to additional warming in the 1990–2018 period, while also reporting larger cumulative emissions expressed as CO_2_-e than expressed as CO_2_-we. At the country level, Gilreath et al. [25] also found that historical (1920–2020) enteric CH_4_ emissions from US beef production were larger when expressed as CO_2_-e than CO_2_-we. Moreover, whereas differences between cumulative CH_4_ emissions were in the 9 to 30% range depending on the level of methodological complexity used (also denoted as tier level by IPCC (2019) [28]), differences between annual emission rates were larger than that for any given year. Finally, Hörtenhuber et al. [24] found a large discrepancy between the climate impact from historical (2005–2019) GHG emissions from Austrian livestock estimated using GWP_100_ and GWP* since annual CH_4_ emission rates decreased over the period examined.

### Projected global warming associated with future emission pathways for global fossil CO_2_, LUC CO_2_, and livestock CH_4_ emissions (2020–2100 period)

**Fig 7:**
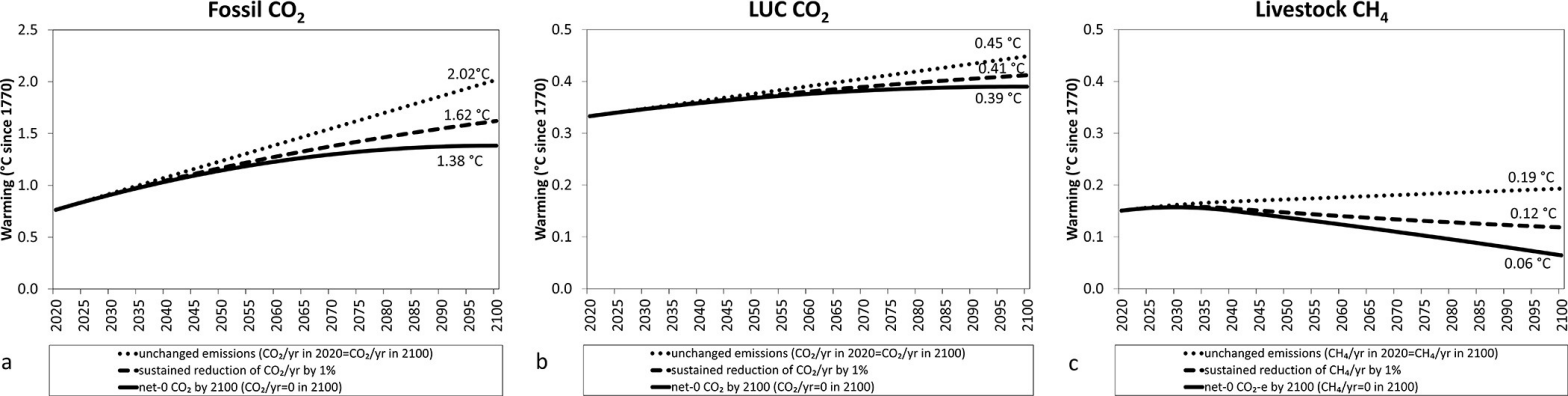
Warming impact of global fossil CO_2_ emissions (a), global land use change (LUC) emissions (b) and livestock CH_4_ (c) as estimated by multiplying aggregated CO_2_ emissions and CO_2_-we (using GWP* for CH_4_) emissions by the TCRE value (0.45°K/Tg CO_2_) since 1770 and considering 3 different future pathways of emissions (2020–2100) (unchanged, reducing 1% per annum, constant over time and reducing emissions gradually until reaching 0 emissions (net-0 CO_2_ or CO_2_-e using GWP_100_ for livestock CH_4_) in 2100: Reducing fossil CO_2_: 0.44 Gt CO_2_/yr; LUC CO_2_: 0.04 Gt CO_2_/yr and livestock CH_4_: 1.5 Mt CH_4_/yr). Note that axes for global fossil CO_2_ is x5 times larger than that for LUC CO_2_ and livestock CH_4_.

Fig 7 shows estimations of warming impacts of future emissions pathways (2020–2100) considering the legacy of warming since the industrial revolution (1770–2019) for 2 global sources of anthropogenic CO_2_ emissions (fossil and LUC) and CH_4_ from enteric fermentation and manure from livestock. Whereas for both CO_2_ sources all pathways, unchanged emissions, reducing emissions at 1% per annum (sustained reduction, constant over time), and reducing emissions to net zero by 2100, result in increased global warming above 2019 levels, only unchanged emissions lead to increased global warming above the 2019 level for CH_4_.

To put this in context, our estimates for historical warming until 2019 associated to CO_2_ from fossil (0.75°C), LUC (0.33°C), and livestock CH_4_ (0.15°C) amounts to 1.23°C for the 3 sources (data in Supplementary Material), which implies that 61%, 27% and 12% of this warming can be attributed to fossil CO_2_, LUC CO_2_, and livestock CH_4_ emissions, respectively. Keeping these sources of emissions unchanged from 2019 until 2100 implies that the relative contribution of fossil CO_2_ to global warming would be much greater (76%) than LUC CO_2_ (17%) and livestock CH_4_ (7%) by 2100. Moreover, keeping these emissions unchanged would result in global warming contributions increasing by 169% (from 0.75°C to 1.38°C), 36% (from 0.33°C to 0.45°C), and 27% (from 0.15°C to 0.19°C) from 2019 levels for fossil CO_2_, LUC CO_2_, and livestock CH_4_, respectively.

Reducing emissions by 1% per annum, constant over time, further reduces the relative contribution to global temperatures above the pre-industrial period of livestock CH_4_ emissions (0.12°C: 6%) compared with fossil CO_2_ (1.62°C, 75%) and LUC CO_2_ (0.41°C, 19%). In fact, reducing livestock CH_4_ emissions by 1% per annum by 2100, would decrease their impact on global warming to levels similar to the early 1990s or a 20% reduction compared with warming levels at 2019 (0.12°C *vs*. 0.15°C). In contrast, a yearly decrease of 1% in CO_2_ emissions would still result in an increase in warming for the year 2100 compared to the existing warming caused in 2019. The warming associated with fossil fuel emissions would increase by 116%, while the warming linked to LUC emissions would rise by 24%.

Reducing all emission rates to zero by 2100 (i.e. CO_2_/yr = 0 or CO_2_-e = 0 for livestock CH_4_) would lead to reversing livestock CH_4_ emissions warming impacts in 2100 to levels observed in the 1940s-1950s, such as a 0.06°C increase relative to 1750 or a 60% reduction relative to 2019. Those warming impacts from livestock CH_4_ emissions would represent 3% of the total warming attributed to the three sources by 2100. Meanwhile, pathways leading to net zero CO_2_ emissions from fossil fuel or LUC would lead to stabilising their impact on global temperatures at 1.38°C (84% relative to 2019 year) and 0.39°C (18% relative to 2019 year) above preindustrial temperatures, respectively. Unlike CO_2_ where it is currently technically possible to reduce net emissions to zero, most technologies for CH_4_ removals are still under development [29]. This limitation for reaching net zero CH_4_ emissions is reflected in the pathways simulated by integrated climate-economic modelling to limit global temperature to 1.5 ^∘^C above pre-industrial times, where biogenic CH_4_ is reduced by 24% to 47% from unspecified sectors relative to 2010, while CO_2_ must be reduced to net-zero ([30]).

These reductions in livestock CH_4_ emissions are also viewed as necessary by Reisinger et al. [19], who argue that future livestock CH_4_ emissions significantly constrain the remaining C budget and the ability to meet stringent temperature limits (i.e. 1.5° C goal). Reisinger et al. [19] advocated for strategies to reduce CH_4_ emissions through more efficient production, technological advances and demand side changes, and importantly always carefully considering their interactions with land-based C sequestration. The challenge is that projected growth in livestock production systems and current CH_4_ emissions from ruminant livestock are greatest in low-and middle-income countries where the nutrient supply to the population could be severely compromised if animal-derived food demand is dramatically shortened. In addition, most production-side mitigation strategies that were designed for developed countries don’t apply to the production systems used in low- and middle-income countries [31,32]. These socioeconomic and environmental aspects of mitigation were ignored in some studies [33] that explored the climate impact of implementing even more drastic scenarios, such as eliminating animal agriculture. Meanwhile, other studies went beyond the livestock sector and explored pathways for global food systems achieving net‑zero emissions by 2050 using GWP* [20] or studied the implications on optimal mitigation options of choosing a particular climate metric for CH_4_’s warming potential [34].

### Projected global warming associated with future global cattle CH_4_ emission pathways leading to a stabilisation of global temperatures by 2050

**Fig 8:**
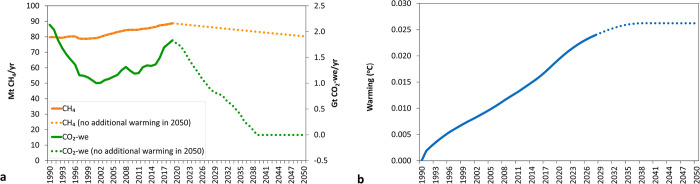
Historical (1990–2019: solid line) and future (2020–2050: dotted line) pathways for global cattle CH_4_ emissions leading to an stabilisation or no additional warming of their impact on global temperature (both historical and future expressed as CH_4_: green line or CO_2_-we using GWP*: orange line) (a) and its corresponding impact on additional temperature caused (blue line, historical: solid line, future: dotted line) as estimated by multiplying aggregated CO_2_-we (using GWP*) emissions by the TCRE value (0.45°K/Tg CO_2_) (b).

Additional warming is stabilised by 2050 at 0.026°C relative to the temperature on 1990 when global cattle CH_4_ emissions are reduced at a sustained annual rate of 0.32% starting in 2020 (Fig 8). This means that CH_4_ emissions greater than a 0.32% annual decrease will contribute to additional warming in the future. This result is consistent with those obtained when applying GWP* to global livestock [20] and US beef [23] CH_4_ emissions pathways and those presented by the GWP* original study [7] where additional warming computed from CO_2_-we was compared with that obtained using a simple climate model.

The sustained decrease in annual global cattle CH_4_ emissions of 0.32% is equivalent to a reduction of annual CO_2_-we emissions from 1.8 Gt in 2019 to 0 in 2050 (green dotted line). These CH_4_ emissions from global cattle would cause additional warming since the reference year (1990) comparable to that from a source of CO_2_ at the rates that are expressed as CO_2_-we. In this case, it would be similar to the emissions of net-zero CO_2_, as depicted by the green line in Fig 8. Altogether, achieving a cumulative reduction of 9.2% by 2050, compared to the year 2020, is necessary for CH_4_ emission rates. This would entail reducing annual emissions from approximately 89 Mt CH_4_/yr (in 2020) to approximately 80 Mt CH_4_/yr by 2050, as indicated by the orange dotted line in Fig 8. Other studies that investigated the impact of different GHG pathways in the livestock sector found dimilar results. Del Prado et al. [27] reported that the European sheep and goat dairy sector could reverse all the warming caused from 2020–2100 to 2020 levels by keeping its production at the level of 2020 and introducing CH_4_ mitigation and C sequestration measures. In another study, Liu et al. [35] showed that the California dairy industry would not contribute additional warming compared to the levels of 1970 in 2030 if CH_4_ emissions can be reduced by 1% per year.

**Fig 9:**
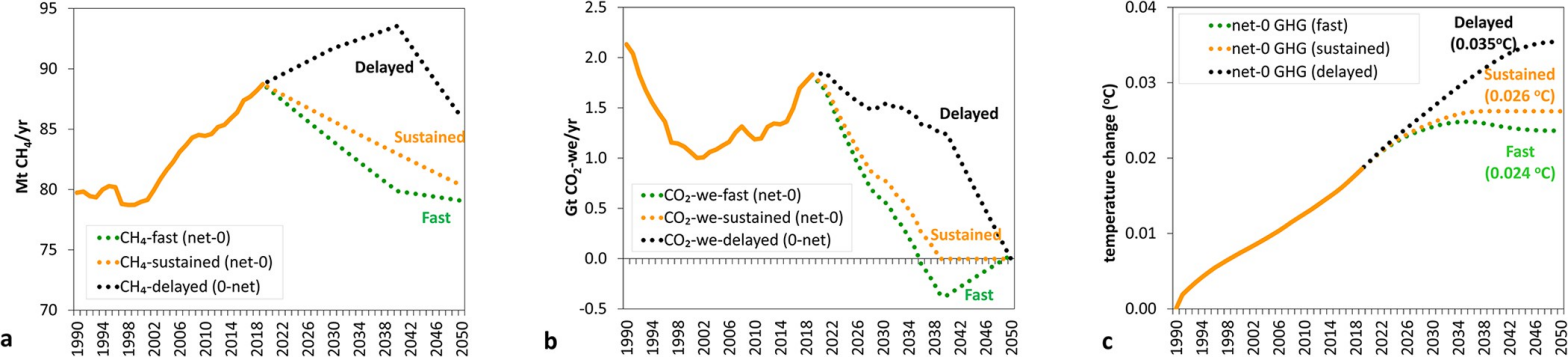
Historical (1990–2019: solid line) and 3 future (2020–2050: dotted line) reduction pathways for global cattle CH_4_ emissions, all leading to a stabilisation of their impact on global temperature (a). These emissions are expressed as CO_2_-we (warming-eq emissions) using GWP* (b) and its corresponding impact on additional temperature caused is estimated by multiplying aggregated CO_2_-we (using GWP*) emissions by the TCRE value (0.45°K/Tg CO_2_) (c). The three future pathways correspond to a ‘fast’ first CH_4_ reductions (dotted green line), ‘sustained’ reductions at 0.32% decrease per year (constant over time) (dotted orange line) and ‘delayed’ introduction of CH_4_ reductions (dotted black line).

Fig 9 compares the global temperature change during the 1990–2050 period by three modelled emission pathways for global cattle CH_4_ emissions that result in no additional warming by 2050 (i.e. CO_2_-we = 0 at the target year of 2050). The three CH_4_ emission reductions pathways as explained in more detail in Materials and Methods section are: 1) ‘fast’ mitigation introduced in 2020, 2) ‘sustained’ mitigation introduced with a fixed reduction rate per year as in Fig 8 (0.32% reduction per year, constant over time), and 3) a ‘delayed’ mitigation strategy introduced in the year 2040 and onwards. Although all three modelled emission pathways lead to stabilising the additional warming caused by CH_4_ emissions by 2050, the pathways that mitigated CH_4_ earlier stabilize warming at a lower (0.024°C) temperature than those that delay CH_4_ mitigation (0.035°C). Del Prado et al. [27] found, for the context of European dairy small ruminants’ systems, that the speed of introduction of mitigation measures makes a considerable near-term impact but a smaller difference by end of century. Essentially, it is not only about stabilising the impact of emissions on additional warming (i.e. reaching zero CO_2_-we), but the rate of change and speed in which CH_4_ mitigation occurs in the reduction pathway that determines the total warming contribution by global cattle CH_4_ emissions by the target date.

## Conclusions

Using the GWP* to express historical global livestock CH_4_ emissions as cumulative CO_2_-we that are converted to temperature change by multiplication with TRCE allows the comparison of global warming emission pathways in an accurate but simpler way than using a climate model [36]. Clearly reductions in global cattle CH_4_ emission rates are needed for their contributions to stop additional warming. A sustained annual reduction of 0.32% would be sufficient to stabilise the effect on global temperatures by global cattle CH_4_ emissions. Conversely, N_2_O and CO_2_ emissions must be eliminated, or equivalent amounts actively removed from the atmosphere (zero emissions), to obtain the same effect on global warming. In fact, as seen with the example for global cattle, reductions in CH_4_ emissions above 0.32% annually would cause analogous effects as increasing C sinks (i.e. active removal of long-lived GHG) and larger reductions would reverse historical temperature impacts from previous decades. As detailed in this study, applying key interventions such as those analysed in [37] to reduce global cattle CH_4_ can even lead to stabilising global temperatures, but the peak temperature achieved would be different and dependent on how quickly and aggressive are the mitigation strategies applied. Moreover, reductions greater than 0.32% annually would partially undo past contributions by global livestock and global cattle to temperature increases, be analogous to active CO_2_ removal from the atmosphere and, depending on the extent of the reduction, approximate to net-zero GHG as defined using GWP_100_.

## Supporting information

S1 File Spreedsheet with all the data used for the figures. (XLSX)
